# The Prevalence of Scoliosis Screening Positive and Its Influencing Factors: A School-Based Cross-Sectional Study in Zhejiang Province, China

**DOI:** 10.3389/fpubh.2022.773594

**Published:** 2022-07-18

**Authors:** Yan Zou, Yun Lin, Jia Meng, Juanjuan Li, Fang Gu, Ronghua Zhang

**Affiliations:** ^1^Zhejiang Provincial Center for Disease Control and Prevention, Hangzhou, China; ^2^The Center for Disease Control and Prevention of Jiaxing City, Jiaxing, China

**Keywords:** scoliosis, screening, children, adolescents, low weight

## Abstract

**Objective:**

Early detection of scoliosis is of great significance to patients with scoliosis and the whole society. This paper aims to learn the prevalence of scoliosis screening positive among students in primary and secondary schools and to explore the influencing factors.

**Methods:**

In 2019, a stratified cluster sampling technique was employed in this school-based cross-sectional study. The sampling covers all prefecture-level cities in Zhejiang Province. Based on the whole class, at least 80 students in each grade of primary school, junior high school, and senior high school were selected. Physical examination and scoliosis screening were performed in the school-based investigation. The distribution of demographic characteristics and nutritional status of children and adolescents with scoliosis screening positive were explored.

**Results:**

A total of 45,547 students were screened. The overall prevalence of children and adolescents with scoliosis screening positive were 3.9%. Higher prevalence of scoliosis screening positive was found in students living in urban area (4.1%), women students (4.1%), students with low weight (5.3%) (*p* < 0.05), and the prevalence increased with age (*p* < 0.05). In logistic analysis, we found age (OR = 1.145; 95% Cis: 1.128, 1.162), gender (OR = 1.118; 95% Cis: 1.016, 1.230) and low weight (OR = 1.48; 95% Cis: 1.25, 1.751) were the influencing factors for prevalence of scoliosis screening positive (*p* < 0.05).

**Conclusions:**

There were no disparities between living areas, but there was a significant difference between genders, among different ages, and among different nutritional statuses of children and adolescents with or without scoliosis screening positive. In multi-analysis, age, gender, and low weight were the influencing factors for the prevalence of scoliosis screening positive. Age and gender-specific scoliosis screening strategies and nutritional public health policies for children and adolescents are needed.

## Introduction

Scoliosis seriously affects the physical and mental health of children and adolescents. Especially in the period of vigorous growth and development, the development of scoliosis deformity is faster. If there is no treatment intervention, the degree of deformity will increase, and the labor capacity will decline. Even there will be cardiopulmonary complications, back pain, and even paraplegia, which will also lead to social and psychological problems (1, 2). Much has yet to be learned about general health, quality of life, and self-image as well as the prevention of students with scoliosis (3). In China, scoliosis is the main type of spinal curvature abnormality, accounting for 82% of spinal curvature abnormality, and a recent study showed the incidence rate for scoliosis was 1.8% (4, 5). Evidence showed that bad posture in adulthood is often formed from childhood, and individuals with severe incorrect posture may be associated with the progress of scoliosis (6). Therefore, the early detection of scoliosis (through school-based screening) is of great significance to patients with scoliosis and the whole society.

Scoliosis screening is an assessment procedure to identify children with spinal curvature at early stages. Many countries have national screening programs for scoliosis in children and adolescents. Scoliosis school screening was first carried out in Delaware in the 1960s and then extended to all parts of the United States, Canada, Europe, and other countries and regions. However, it is controversial whether to implement compulsory screening (7). The United States systematically evaluated Adolescent Idiopathic Scoliosis (AIS) screening, including 14 studies covering 448,276 people, and found that screening can detect AIS. Bracing and possibly exercise treatment can interrupt or slow the progression of curvature in adolescence (8). Australia supports scoliosis screening in schools to facilitate early treatment (7). The study in Denmark confirms that in a health care system without school screening, patients with AIS referred for evaluation by general practitioners have larger curve sizes compared to systems with school screening (9). A population-based study with a long-term follow-up in Singapore indicated that a scoliosis screening program can have sustained clinical effectiveness in identifying patients with adolescent idiopathic scoliosis needing clinical observation. As the prevalence of adolescent idiopathic scoliosis increases, scoliosis screening should be continued as a routine health service in schools or by general practitioners if there is no scoliosis screening policy (10). In recent years, some regions in China have carried out epidemiological surveys of scoliosis, but scoliosis screening has not been included in the physical examination of primary and secondary schools by governments and education departments (11). In addition, there were few scoliosis investigations carried out domestically, and in the investigation carried out, due to the differences in screening methods, it is difficult to compare and evaluate each other. By the limited investigation in China, the influencing factors of scoliosis screening positive have not been explored and analyzed. This paper aims to learn the prevalence of scoliosis screening positive among students and its influencing factors.

## Methods

### Study Design and Data Sources

There are 6,000 primary and secondary schools in Zhejiang Province. Stratified cluster sampling methods were used in this study. In 2019, sampling covers all prefecture-level cities in Zhejiang Province. Samples were stratified into urban areas and rural areas. In each prefecture-level city, seven schools in the urban area (two primary schools, two junior high schools, two senior high schools, and one vocational high school), five schools in the rural area (two primary schools, two junior high schools, and one senior high school) were selected by the method of random sampling. Then, based on the whole class, at least 80 students in each grade of primary school, junior high school, and senior high school were selected by the method of a random sampling of the whole class, that is, at least 480 students were selected from each primary school and 240 students were selected from each junior high school, senior high school.

All methods were performed in accordance with the relevant guidelines and regulations, and this study was approved by the Ethics Committee of the Zhejiang Provincial Center for Disease Control and Prevention (T-043-R20180515). The parents or guardians of the participants provided consent to participate on behalf of the minors.

### Screening of Spinal Curvature Abnormality

Physical examination and scoliosis screening were performed in the school-based investigation by trained doctors and nurses from local community health centers. Boys and girls were examined separately. Scoliosis screening and evaluation were based on the screening of spinal curvature abnormality in children and adolescents (GB/T 16133-2014). The exclusion criteria for this study were as follows: students who are unable to perform Adam's forward bending test due to any reasonable cause. General examination for scoliosis screening includes an examination of shoulder asymmetry, scapula prominence, unequal waistline or arm span, and abnormalities involving the trunk or spine, such as humps in the ribs or lumbar regions. Thereafter, Adam's forward bending test was used to screen for scoliosis in the population. The measurement was performed in the standing position during forwarding bending of the trunk (12). Evaluation of scoliosis (screening positive or negative) was based on the result of the general examination combined with Adam's forward bending test.

### Anthropometrical Measurement and Data Collection

Children and adolescents' sex and age were collected by general information questionnaire. Anthropometrical measurements were conducted by well-trained health workers of local community health centers. Height was measured without shoes to the nearest 0.2 cm using a portable SECA stadiometer, and weight was measured without shoes and in light clothing to the nearest 0.1 kg on a calibrated beam scale. Body mass index (BMI) was calculated as weight (kg)/height (m)^2^. According to “Screening for overweight and obesity among school-age children and adolescents” (13), we judged overweight and obesity of children aged 6–17 years old by boundary-value. According to “Screening standard for malnutrition of school-age children and adolescents” (14), we judged the low weight of children aged 6–17 years old.

### Statistical Analysis

All the data were analyzed with SAS9.4 (SAS Institute Inc, Cary, NC, USA). Category variables were present in the form of numbers and percentages. The Chi-square test was used to explore the associated factors of scoliosis screening positive. Multiple logistic regression was used to analyze the influencing factors of the prevalence of scoliosis screening positive.

A two-side *p* < 0.05 was considered statistically significant.

## Results

### Subject Characteristics

A total of 45,547 students aged 6–17 were investigated and screened with scoliosis, including 23,706 men and 21,841 women. The overall prevalence of scoliosis screening positive among students was 3.9%. The prevalence of low weight, overweight, and obesity among the participants were 6.6, 14.3, and 12.4%, respectively.

### Prevalence by Age and Living Area

Urban areas can include towns and cities. The prevalence of scoliosis screening positive among children and adolescents in urban areas was 4.1%, which was higher than that in rural areas (*p* < 0.05; Table 1). Especially, the difference existed among children and adolescents aged 7, 11, 15, 16, and 17 years old (*p* < 0.05).

**Table 1:** The prevalence of scoliosis screening is positive among students stratified by the living area.

**Age**	**Urban area**			**Rural area**			**Chi-Square**	***p***
	***N***	**Scoliosis screening positive**	**Positive rate %**	***N***	**Scoliosis screening positive**	**Positive rate %**		
6	1,672	16	1.0	1,669	24	1.4	1.634	>0.05
7	1,911	16	0.8	1,934	55	2.8	21.354	<0.001
8	1,948	29	1.5	1,867	42	2.2	3.022	>0.05
9	1,842	36	2.0	1,808	53	2.9	3.661	>0.05
10	1,984	41	2.1	2,036	58	2.8	2.559	>0.05
11	1,930	50	2.6	1,999	85	4.3	8.17	<0.01
12	1,901	85	4.5	1,950	106	5.4	1.9	>0.05
13	1,906	122	6.4	1,998	100	5.0	3.544	>0.05
14	1,992	116	5.8	1,996	103	5.2	0.844	>0.05
15	2,734	167	6.1	1,171	51	4.4	4.78	<0.05
16	2,785	168	6.0	905	24	2.7	15.824	<0.001
17	2,712	184	6.8	897	35	3.9	9.828	<0.01
Total	25,317	1,030	4.1	20,230	736	3.6	9.394	<0.01

**Figure 1:**
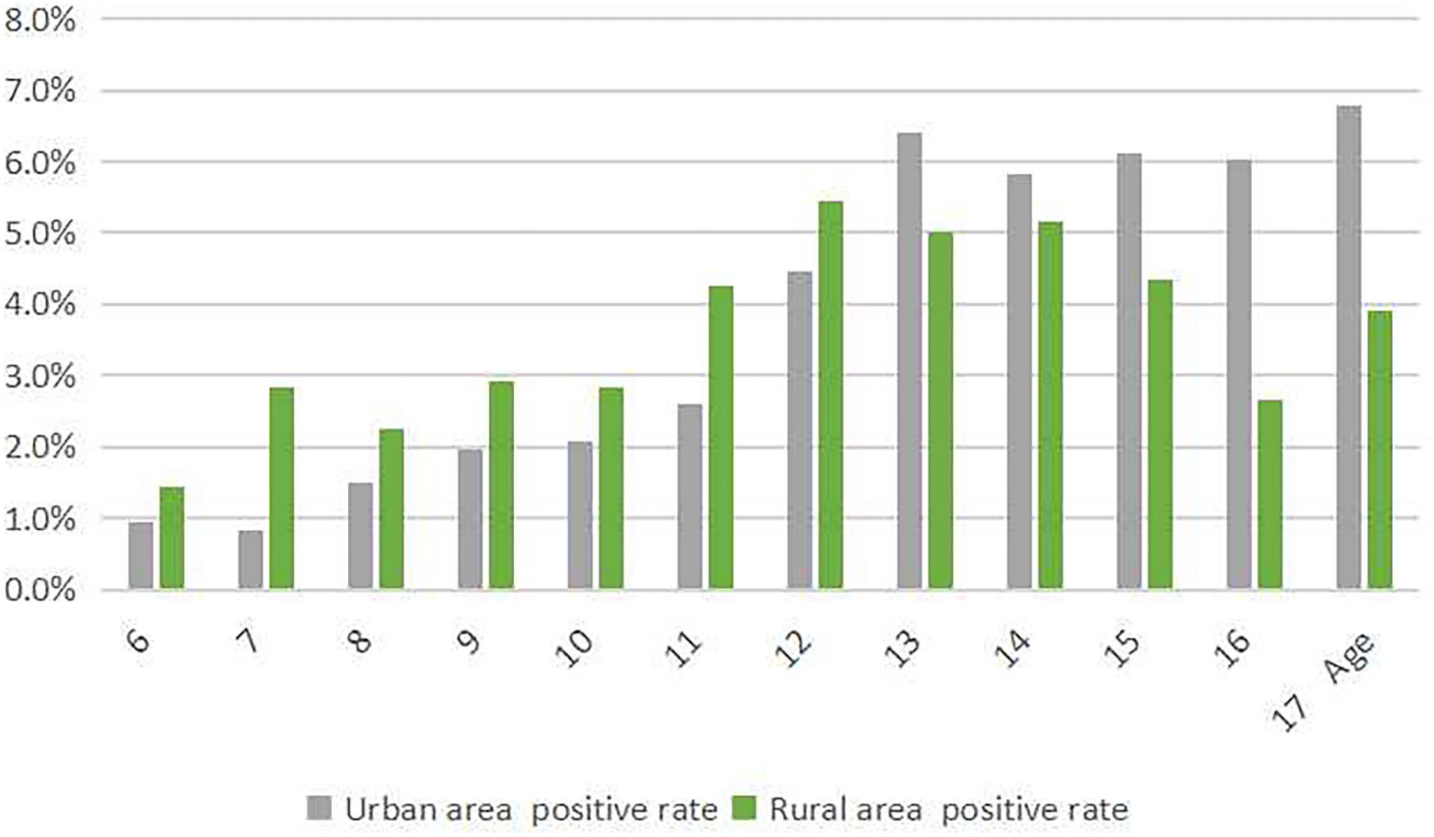
The prevalence of scoliosis screening positive among children and adolescents stratified by the living area.

In urban areas, the prevalence of scoliosis screening positive was 6.8% among children and adolescents aged 17, while in rural areas, the prevalence was 5.4% among children and adolescents aged 12, both higher than that of other ages, respectively (*p* < 0.05; Figure 1).

### Prevalence by Age and Gender

The prevalence of scoliosis screening positive among children and adolescents who are women was 4.1%, which was higher than that of men (*p* < 0.05; Table 2). Especially, the difference existed among children and adolescents aged 8, 10, 11, and 16 years old (*p* < 0.05).

**Table 2:** The prevalence of scoliosis screening is positive among students stratified by gender.

**Age**	**Male**			**Female**			**Chi-Square**	***p***
	***N***	**Scoliosis screening positive**	**Positive rate %**	***N***	**Scoliosis screening positive**	**Positive rate%**		
6	1,718	22	1.3	1,648	18	1.1	0.208	>0.05
7	2,020	39	1.9	1,825	32	1.8	0.166	>0.05
8	2,019	27	1.3	1,796	44	2.4	6.442	<0.05
9	1,951	45	2.3	1,674	44	2.6	0.306	>0.05
10	2,071	34	1.6	1,949	65	3.3	11.986	<0.01
11	2,051	48	2.3	1,878	87	4.6	15.525	<0.001
12	1,957	94	4.8	1,894	97	5.1	0.207	>0.05
13	2,027	127	6.3	1,877	95	5.1	2.635	>0.05
14	2,005	99	4.9	1,983	120	6.1	0.383	>0.05
15	2,050	106	5.2	1,855	112	6.0	1.389	>0.05
16	1,932	114	5.9	1,758	78	4.4	3.998	<0.05
17	1,905	121	6.4	1,704	98	5.8	0.569	>0.05
Total	23,706	876	3.7	21,841	890	4.1	5.38	<0.05

**Figure 2:**
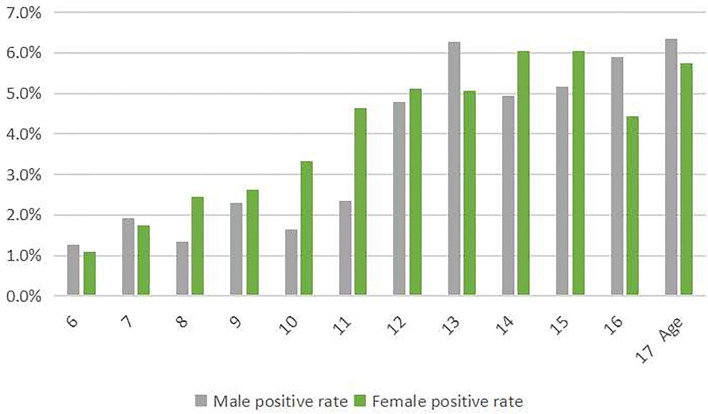
The prevalence of scoliosis screening positive among children and adolescents stratified by gender.

The prevalence of scoliosis screening positively increased with age both in men and women (*p* < 0.05; Figure 2). When we divided age into two groups (6–10 and 11–17 years old), we found that the prevalence of scoliosis screening positive of students aged 11–17 was higher than that aged 6–10 (χ^2^ = 305.261, *p* < 0.001).

### Prevalence by Age and Nutritional Status

We classified the nutritional status as low weight, eutrophic (normal BMI), overweight, and obese. According to “Screening for overweight and obesity among school-age children and adolescents,” we judged overweight and obesity. According to “Screening standard for malnutrition of school-age children and adolescents,” we judged low weight. The prevalence of scoliosis screening positive among children and adolescents with low weight was 5.3%, which was higher than that without low weight (*p* < 0.05; Table 3). Especially, the difference existed among children and adolescents aged 9, 10, 11, 12, 13, 14, 15, 16, and 17 years old (*p* < 0.05).

**Table 3:** The prevalence of scoliosis screening is positive among students stratified by nutritional status.

	**Low weight**			**Eutrophic**			**Overweight**			**Obesity**			**Chi-Square**	***p***
	***N***	**Scoliosis screening positive**	**Positive rate %**	***N***	**Scoliosis screening positive**	**Positive rate %**	***N***	**Scoliosis screening positive**	**Positive rate %**	***N***	**Scoliosis screening positive**	**Positive rate %**		
6	192	2	1.0	2,161	27	1.2	476	8	1.7	512	3	0.6	2.918	>0.05
7	327	2	0.6	2,480	53	2.1	529	9	1.7	509	7	1.4	4.592	>0.05
8	354	6	1.7	2,480	54	2.2	450	7	1.6	531	4	0.8	6.183	>0.05
9	252	13	5.2	2,333	55	2.4	503	9	1.8	562	12	2.1	9.011	<0.05
10	242	6	2.5	2,589	75	2.9	446	10	2.2	743	8	1.1	8.064	<0.05
11	253	11	4.3	2,480	96	3.9	655	20	3.1	541	8	1.5	8.583	<0.05
12	226	16	7.1	2,567	139	5.4	585	24	4.1	473	12	2.5	11.085	<0.05
13	195	25	12.8	2,634	159	6.0	624	24	3.8	451	14	3.1	28.654	<0.001
14	211	19	9.0	2,746	162	5.9	606	21	3.5	425	17	4.0	12.514	<0.01
15	228	11	4.8	2,730	176	6.4	594	21	3.5	353	10	2.8	13.904	<0.01
16	241	22	9.1	2,600	137	5.3	542	25	4.6	307	8	2.6	12.134	<0.01
17	277	26	9.4	2,561	157	6.1	490	30	6.1	281	6	2.1	12.996	<0.01
Total	2,998	159	5.3	30,361	1,290	4.2	6,500	208	3.2	5,688	109	1.9	7.739	<0.05

**Figure 3:**
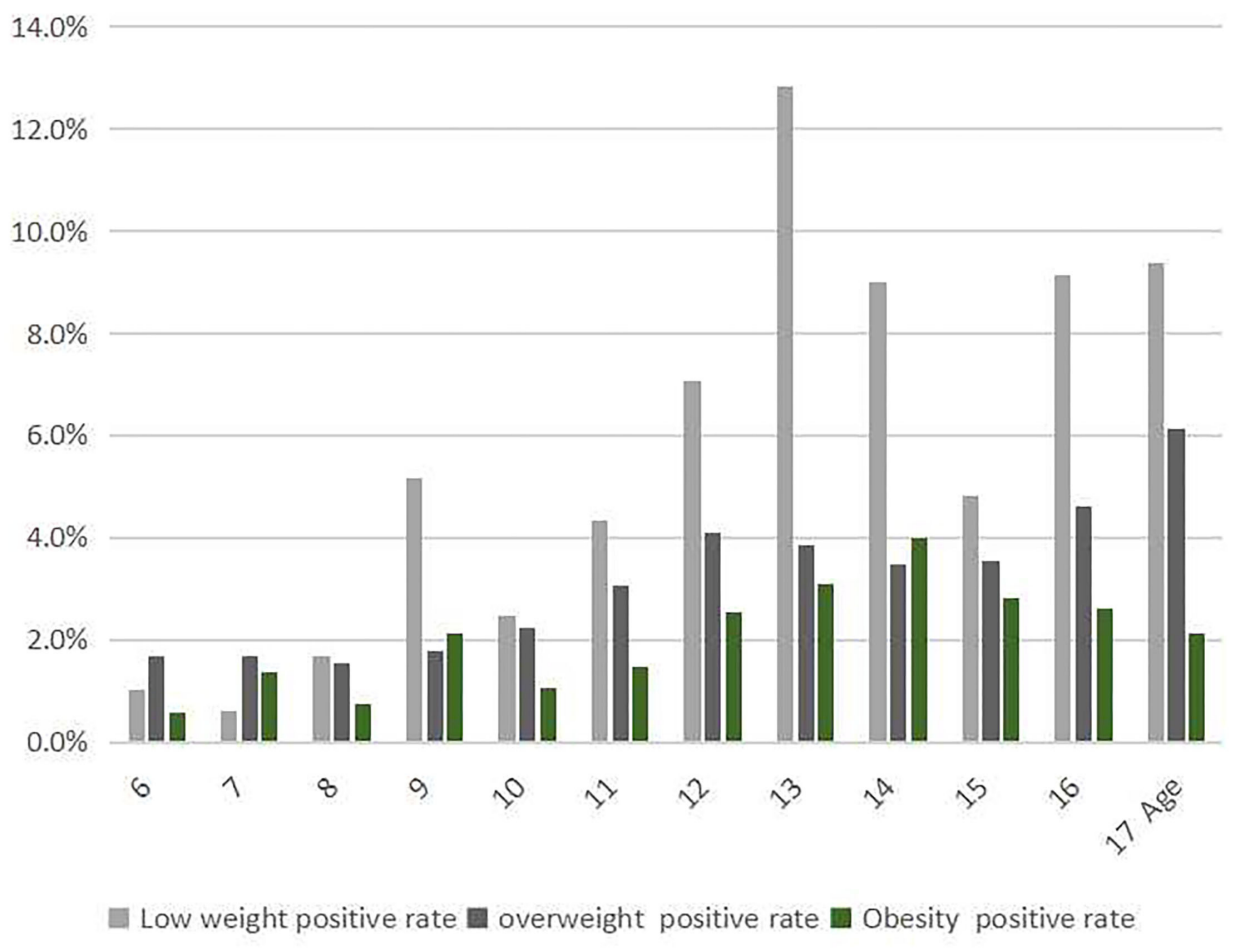
The prevalence of scoliosis screening positive among children and adolescents stratified by nutritional status.

The prevalence of scoliosis screening positive was 12.8% among children and adolescents aged 13 with low weight, while that was 4% among children and adolescents aged 14 with obesity, both higher than that of other ages, respectively (*p* < 0.05; Figure 3).

### Influencing Factors for the Prevalence

In multi factor regression analysis, we found age (OR = 1.145, 95% CI: 1.128, 1.162), gender (OR = 1.118, 95% CI: 1.016, 1.23), and low weight (OR = 1.48, 95% CI: 1.25, 1.751) were the influencing factors for prevalence of scoliosis screening positive (*p* < 0.05; Table 4).

**Table 4:** Logistic analysis of influencing factors for prevalence of scoliosis screening positive among students.

**Variables**	**Wald c^2^**	**p**	**OR**	**95% C.I. for EXP(B)**	
				**Lower**	**Upper**
Age	306.651	<0.001	1.145	1.128	1.162
Gender (Male/Female)	5.203	<0.05	1.118	1.016	1.230
Living area (Urban area/ Rural area)	0.606	>0.05	1.040	0.942	1.148
Low weight (Yes/No)	20.784	<0.001	1.480	1.250	1.751

## Discussion

The present study illustrates the prevalence of scoliosis screening positive among students and compared the disparities between genders, living areas, different ages, and different nutritional statuses in students with or without scoliosis screening positive. We found age, gender, and low weight were the influencing factors for the prevalence of scoliosis screening positive.

In this study, we found the prevalence of scoliosis screening positive among students was 3.9%, which was lower than a report from Guangzhou with 6.56% (15), while higher than a report from Wuxi with 2.6% (16). Our study covers primary and junior high school students. There are other studies that found different prevalence because of the differential ages of the subjects. And, because the sampling method, will also lead to the bias caused by selection. Another study also reported that screening of 13–15 years old girls identified a significant number who could benefit from preventive treatment (13). Penha also reported that the prevalence of scoliosis was higher during puberty years of age (17). Fong reported that a scoliosis screening program can have sustained clinical effectiveness in identifying patients with scoliosis needing clinical observation (11). Scoliosis typically develops in late childhood and more than 4% of adolescents between the ages of 11–17 years old showing spinal malformation (17, 18). The Scoliosis Research Society recommends screening children and adolescents annually to prevent spinal deformation and identifies symptoms at an early stage (18). Similar to these works of literature, we found that the prevalence of scoliosis screening positive in students aged 11–17 was higher than that aged 6–10. This founding suggests that in areas without mandatory screening and with limited resources, we should prefer to provide screening services for students aged 11–17 years.

Female students should be paid more attention. A previous study in China reported that girls had a higher prevalence in each age subgroup compared with boys (19). Another study carried out on Chongming Island (China) found the prevalence was 2.52 %, which was higher in girls (3.11%) than in boys (1.96%) (20). Similar to the previous studies, we found that the prevalence of scoliosis screening positive among female students was higher than that of men students. Women students with low BMI may have lower leptin levels, this may be one of the reasons for the higher scoliosis prevalence among female students. Adamczewska et al. reported that age, sex, and risk of developing angle of trunk rotation are very closely associated (21), suggesting that females may have different scoliosis progress and prognosis compared with males.

We also found that the prevalence of scoliosis screening positive among students in urban areas was higher than that in rural areas. But in the multi-factor analysis, the influence of living area had no significant effect on the prevalence of scoliosis screening positive. Students with a scoliosis screening positive must have more probability to be diagnosed with scoliosis after they were referred to the hospital. Golboni et al. reported the rural area residents suffered more from low functional health literacy, compared with their urban counterparts (22). Health literacy is lower in the rural population although this difference is explained by known confounders, and community and societal level factors should be focused on promoting health care (23, 24). Scoliosis is a serious clinical problem that requires systematic physical therapy and control of body balance-treatment from the moment of achieving skeletal maturity in a child. In general, children and adolescents living in urban areas could benefit from health literacy and receive more attention to healthy behavior and lifestyles from their families, so they are more likely to go to the hospital for screening and be referred to the hospital if there are abnormal conditions. Latalski et al. reported there is a relationship between the economic standard of the family and engagement in the treatment of a child with scoliosis (25). So, the health education for the children and adolescents who are screened positive, especially the referral and thereafter interventions for those living in the rural area, should be paid more attention to.

A system review demonstrates that patients with AIS are significantly more likely to have a low BMI compared to the general population (26). Sun et al. reported that low body mass index can be predictive of bracing failure in patients with adolescent idiopathic scoliosis (27). Xu et al. reported that BMI was an important indicator of pulmonary function in scoliosis patients (28). Low BMI is not only related to treatment and intervention, but also to scoliosis prevalence. Kim et al. reported that the prevalence of thin students with scoliosis could increase by up to four times depending on the BMI criteria (29). Jeon and Kim reported that low weight and the risk of developing scoliosis are very closely associated (30). A study in China found that compared with healthy students, those with scoliosis were taller, had lower weight, and lower BMI (5). Similar to these works of literature, we found the prevalence of scoliosis screening positive was high in students with low weight. Also, in this study, we found the prevalence of low weight among students was 6.6%. However, with the rapid economic transformation and development in recent years, more attention has been paid to child obesity, while the problem of low weight has been overlooked. Reasonable diet and balanced nutrition should be advocated, and maintenance of appropriate and normal weight should be encouraged to prevent and reduce the risk of scoliosis.

In addition, the methodological limitations of the study are that SAS software is not able to analyze the interactions between factors on the response variable, so the interaction effects of age, gender, weight, and other independent variables on the scoliosis screening positive should be explored in further studies.

## Conclusions

The prevalence of scoliosis screening positive among students was 3.9% in this study. There were no disparities between living areas, but there was a significant difference between genders, among different ages, and among different nutritional statuses of children and adolescents with or without scoliosis screening positive. In multi-analysis, age, gender, and low weight were the influencing factors for the prevalence of scoliosis screening positive. Age and gender-specific scoliosis screening strategies and nutritional public health policies for children and adolescents are needed.

## Data Availability Statement

The raw data supporting the conclusions of this article will be made available by the authors, without undue reservation.

## Ethics Statement

The studies involving human participants were reviewed and approved by the Ethics Committee of Zhejiang Provincial Center for Disease Control and Prevention. Written informed consent to participate in this study was provided by the participants' legal guardian/next of kin.

## Author Contributions

YZ and RZ: conceptualization and resources. YZ and YL: methodology. JM and FG: software. JL and JM: investigation. YZ: writing and project administration. All authors read and approved the final manuscript.

## Funding

This work was supported by the Basic Public Welfare Research Plan of Zhejiang Province (LGF19H260002).

## Conflict of Interest

The authors declare that the research was conducted in the absence of any commercial or financial relationships that could be construed as a Potential Conflict of Interest.

## Publisher's Note

All claims expressed in this article are solely those of the authors and do not necessarily represent those of their affiliated organizations, or those of the publisher, the editors and the reviewers. Any product that may be evaluated in this article, or claim that may be made by its manufacturer, is not guaranteed or endorsed by the publisher.
